# Graft versus host disease following liver transplantation: A case report

**DOI:** 10.3892/etm.2014.1850

**Published:** 2014-07-17

**Authors:** CHANGSONG ZHANG, GUANGSHUN YANG, YANG LING, GUIHUA CHEN, TIANBAO ZHOU

**Affiliations:** 1 Clinical Oncology Laboratory, Changzhou Tumor Hospital, Medical College of Soochow University, Changzhou, Jiangsu 213002, P.R. China; 2 Hepatic Surgery Center, Eastern Hepatobiliary Surgery Hospital, Second Military Medical University, Shanghai 200438, P.R. China; 3 Hepatic Surgery Center, The Third Affiliated Hospital, Sun Yat-sen University, Guangzhou, Guangdong 510630, P.R. China; 4 Hepatobiliary Surgery Center, The Affiliated Ningbo No. 2 Hospital, Ningbo University School of Medicine, Ningbo, Zhejiang 315010, P.R. China

**Keywords:** graft versus host disease, liver transplantation

## Abstract

Graft versus host disease (GVHD) is an uncommon complication following liver transplantation. In the present case report, a 53-year-old male hepatitis B virus carrier was diagnosed with primary liver cancer with post-hepatitis cirrhosis. Preoperative cytomegalovirus (CMV), Epstein-Barr virus, coxsackievirus, herpes simplex virus and autoimmune antibody series were negative. Preoperative human leukocyte antigen type was also negative. Following classic orthotropic liver transplantation, postoperative treatment included immunosuppression therapy, infection protection, anti-human immunodeficiency virus therapy and CMV infection protection therapy. Chemotherapy was initiated at day 16 following surgery. At day 26 following the transplantation, the patient developed a fever of unknown cause, and a scattered red rash was observed behind the left ear and on the neck. The patient presented with a fever of unknown cause, rash, symptoms of the digestive tract, leukocytopenia and pancytopenia. A diagnosis of GVHD was confirmed following a skin biopsy. Symptomatic therapies, including antivirals, anti-anaphylaxis drugs and steroids were administered. However, the patient succumbed to infection, acute respiratory distress syndrome and multiple organ failure at day 46 following surgery. Therefore, an effective therapeutic strategy for the treatment of GVHD following liver transplantation is yet to be established, and further research is required prior to such a regimen being developed.

## Introduction

Graft versus host disease (GVHD) is an immunoreaction that occurs when implanted T lymphocytes from the donor recognize cell surface antigens in the host and mediate cytotoxicity (1). GVHD, classified as an acute and chronic disease, is rarely observed following liver transplantation. Post-liver transplantation GVHD was first reported in 1987; however, the underlying mechanisms remain to be elucidated, although it is considered to be associated with the transplantation of T lymphocytes from the donor.

The development of this type of GVHD is divided into three stages. Firstly, the pre-treatment stage where chemotherapy, release of endotoxins following infection, blood transfusion, prior treatment and underlying diseases that affect endothelial and epithelial cells operate to induce the release of inflammatory cytokines, including interleukin (IL)-1, IL-6 and tumor necrosis factor (TNF)-α. This upregulates the expression of antigens and adhesion molecules on histocytes of target organs, including the skin, gastrointestinal mucosa and liver; thus, an immunoreaction with T lymphocytes of the donor occurs. The second stage is classified by the activation of donor T lymphocytes. Inconsistency of major histocompatability complex (MHC)-I antigens of the donor and recipient results in CD8^+^ cytotoxic T cell proliferation, while inconsistency of MHC-II antigens results in CD4^+^ T cell proliferation. Furthermore, T cells become polarized into CD4^+^ T helper1 cells that secrete IL-2 and interferon (IFN)-γ, which in turn cause T cell proliferation and the activation of natural killer cells. Finally, GVHD occurs when IL-2 and IFN-γ activate donor mononuclear cells to produce large amounts of inflammatory cytokines, including IL-2 and TNF-α (2). The ‘cytokine-injury-cytokine’ cycle causes a waterfall-like development of inflammatory cytokines, which ultimately results in the clinical presentation of GHVD (3).

A total of 587 patients underwent an orthotopic liver transplantation between May 2003 and October 2008 in the Eastern Hepatobiliary Hospital (Shanghai, China); however, only one case developed postoperative GVHD, which is reported in the present study. The study was approved by the Ethics Committee of the Eastern Hepatobiliary Surgery Hospital of the Second Military Medical University (Shanghai, China) and informed consent was provided by the patient’s family.

## Case report

A 53-year-old male patient was admitted to the Eastern Hepatobiliary Hospital due to a space occupying lesion of the right liver observed by physical examination 20 days previously. A computed tomography scan revealed disproportion of the liver lobes and a 4-cm low-density focus in the right posterior liver lobe. Preoperative cytomegalovirus (CMV), Epstein-Barr virus (EBV), coxsackievirus, herpes simplex virus and autoimmune antibody series were negative. Preoperative human leukocyte antigen type was also negative. The patient tested positive for hepatitis B surface antigen, hepatitis B e antibody and hepatitis B c antibody, and had an α-fetoprotein level of 288,810 μg/l and B type blood. Liver function, biochemistry and blood routine tests were within the normal range. The patient was diagnosed with primary liver cancer, post-hepatitis cirrhosis and was found to be a hepatitis B virus carrier. On September 27, 2007, the patient received a classic orthotropic liver transplantation. Intraoperative blood loss was 400 ml and a blood transfusion was not administered. Methylprednisolone (500 mg) was administered intravenously and 4,000 IU hepatitis B immunoglobulin (HBIG) was administered intramuscularly. Postoperative treatment included: (i) Immunosuppression therapy consisting of FK506, CellCept and methylprednisolone (within 7 days following surgery), and prednisone tablets instead during the later stages; (ii) infection protection and anti-human immunodeficiency virus therapy comprising rocephin, tuinidazole and daily intramuscular injections of 1200 IU HBIG (dosage was adjusted according to the blood drug concentration, but usually maintained at >500 units); and (iii) CMV infection protection therapy consisting of ganciclovir (within 14 days following surgery) and aciclivir. Chemotherapy was initiated at day 16 following surgery using a fluorouracil, mitomycin and cisplatin protocol for six days.

The patient recovered smoothly within three weeks following surgery, and the transplanted liver function was normal. The FK506 concentration was maintained at 8–12 ng/ml. At day 26 following surgery, the patient developed a fever (38.2°C) of unknown cause. At day 27, a scattered red rash was observed behind the left ear and on the neck. Laboratory analysis at day 33 revealed that the white blood cell (WBC) count was 3.74×10^9^, the red blood cell count was 2.65×10^12^, the platelet count was 83×10^9^, the concentration of blood urea nitrogen (BUN) was 11.8 mmol/l, the creatine level was 96 μmol/l and liver function was near the normal value. The patient had yellow watery diarrhea at day 34 following surgery, and continued to have a fever of unknown cause, a rash, symptoms of the digestive tract, leukocytopenia and pancytopenia. The patient’s temperature fluctuated between 37.4 and 39°C. No positive bacteria were found in repeated blood, sputum and urine cultures during this period, and tests for Merkel cell polyomavirus antigens and EBV antibodies were negative. A bile culture revealed hemolytic staphylococcia. The rash exhibited raised red macular eruptions, tenderness and bleaching on compression. A rash was observed at day 36 following surgery, and appeared (in order) on the neck, chest, back, abdomen and four extremities, and exfoliated in the same order. Skin color became normal following exfoliation. The digestive symptoms were comparatively mild, presenting as nausea and vomiting, watery diarrhea and mild oral ulceration. The lowest peripheral blood count was 0.07×10^9^/l for WBCs and 11×10^9^/l for platelets. Liver function was near the normal value and BUN of renal function was slightly higher compared with the normal value.

**Figure 1:**
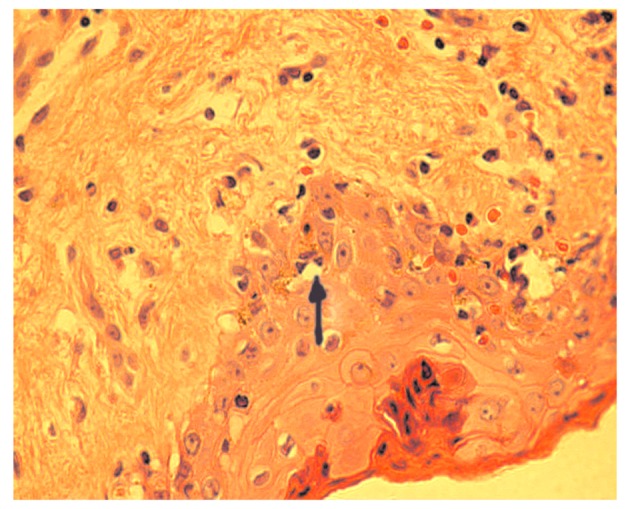
A skin biopsy was performed six days following the appearance of symptoms (33 days following surgery).

Diagnoses of viral infection, acute rejection reaction, drug rash and immunosuppressant toxicity were suspected when the patient first developed the symptoms; however, a skin biopsy six days following the appearance of the symptoms (33 days following surgery) supported the skin pathological presentation of acute GVHD. Thus, a diagnosis of GVHD was confirmed (Fig. 1). Prior to the consideration of GVHD, symptomatic therapies, including antivirals, anti-anaphylaxis drugs and steroids, were administered, while FK506 was maintained at 8–12 ng/ml. Following the diagnosis of GVHD, the steroid dosage was at a maximum (initial dosage of methylprednisolone was 40 mg/day q.d., which was gradually decreased to 10 mg/day q.d. and 20 mg/day q.d.). Filgrastim, recombinant IL-11 and Fufang Zaofan Wan were administered when bone marrow arrest was present and ganciclovir was discontinued. The rash and digestive symptoms were relieved following treatment; however, the fever and peripheral blood counts remained unchanged. The patient succumbed to infection, acute respiratory distress syndrome and multiple organ failure at day 46 following the transplant surgery.

## Discussion

Acute GVHD occurs following transplantation (within 100 days), usually between three and five weeks. In the present case, the initial symptoms of GVHD started at day 26 following liver transplantation. A diagnosis of acute GVHD depends firstly on clinical presentation, including fever, rash, diarrhea and severe neutropenia or pancytopenia, with normal or almost normal liver function and slightly abnormal renal function. Secondly, the diagnosis depends on skin pathological observations, including the epithelial segment becoming loose and keratinous, the spinous layer becoming thinner and atrophic, the skin processes disappearing, focal necrotic inflammatory scabs in the upper spinous layer and necrosis of single or aggregated keratinized cells in the inner spinous layer. The mortality rate of acute GVHD is high, and only a few cases of successful treatment have been reported (4). In the present case report, the onset symptoms were a fever and rash, followed by bone marrow suppression, leading to neutropenia and an increased risk of infection. Finally, the patient succumbed to an infection and associated multiple organ failure. Lymphocytic infiltration was observed in the majority of the chromophils of the shallow dissection. Acute GVHD primarily involves the skin, gastrointestinal tract and liver since these tissues proliferate more actively; thus, are more liable to express MHC-I and MHC-II molecules and contain antigen presenting cells from hematopoietic cells. According to Keystone criteria (5), acute GVHD is clinically classified into four stages: Stage I, rash area of <25%, hemoglobin (Hb) level of 24.2–51.3 μmol/l and diarrhea of >500 ml or persistent nausea; stage II, rash area of 25–50%, Hb level of 51.3–102.6 μmol/l and diarrhea of >1,000 ml or persistent nausea; stage III, rash area of >50%, Hb level of 102.6–256.5 μmol/l and diarrhea of >1,500 ml; and stage IV, extensive erythrodermia accompanied with the formation of blisters, Hb level of >256.5 μmol and severe abdominal pain with or without intestinal obstruction (6).

Controversy remains over the occurrence of acute GVHD; however, it is agreed that the prognosis is poor and at present, there is no established effective therapeutic strategy. For post-liver transplantation GVHD, immunosuppressants in combination with 2–2.5 mg/kg/day methylprednisolone are administered clinically. Between 1 and 20 mg/kg/day methylprednisolone is recommended as the initial dosage, and the dosage of steroids should be reduced when the condition is controlled. However, in the majority of cases, the condition exacerbates following a reduction in medication; the mortality rate from which is only next to that from opportunistic infection. The initial dose of methylprednisolone administered in the present case was low and the condition was not controlled effectively. However, increasing the dose of methylprednisolone increases the opportunity of fungal infection. Combined use of infliximab and pentostatin may avoid long-term sequelae from the use of steroids (7). Previous observations indicate that tacrolimus is better than ciclosporin in preventing the occurrence of stage III and IV acute GVHD (8). The pharmacology of tacrolimus and ciclosporin is similar, but the former is a more potent calcium-mediated phosphotase inhibitor; thus, the dosage can be lower. Mycophenolate mofetil inhibits the synthesis of RNA and DNA by inhibiting hypoxanthine mononucleotide dehydrogenase. As mycophenolate mofetil has a key role in interfering with lymphocytes in stage I and II acute GVHD, this drug has attracted increasing attention (9). In the present case, mycophenolate mofetil was discontinued very early with the consideration that it may facilitate pancytopenia. Sirolimus has an important role in preventing GVHD. Unlike ciclosporin and tacrolimus, sirolimus inhibits mammalian target of rapamycin, which is essential for T cell proliferation; thus, halting the cell cycle (10). Theoretically, using a drug regimen that may prevent the three stages from occurring is best; however, further research is required prior to such a regimen being developed.

## References

[1] Thin L, Macquillan G, Adams L (2009). Acute graft-versus-host disease after liver transplant: novel use of etanercept and the role of tumor necrosis factor alpha inhibitors. Liver Transpl.

[2] Christopeit M, Schütte V, Theurich S, Weber T, Grothe W, Behre G (2009). Rituximab reduces the incidence of acute graft-versus-host disease. Blood.

[3] D’Asaro M, Salerno A, Dieli F, Caccamo N (2009). Analysis of memory and effector CD8^+^ T cell subsets in chronic graft-versus-host disease. Int J Immunopathol Pharmacol.

[4] Rezvani AR, Storb RF (2012). Prevention of graft-vs. -host disease. Expert Opin Pharmacother.

[5] Przepiorka D, Weisdorf D, Martin P, Klingemann HG, Beatty P, Hows J, Thomas ED (1995). 1994 consensus conference on acute GVHD grading. Bone Marrow Transplant.

[6] Foley JE, Jung U, Miera A (2005). Ex vivo rapamycin generates donor Th2 cells that potently inhibit graft-versus-host disease and graft-versus-tumor effects via an IL-4-dependent mechanism. J Immunol.

[7] Wilson J, Cullup H, Lourie R (2009). Antibody to the dendritic cell surface activation antigen CD83 prevents acute graft-versus-host disease. J Exp Med.

[8] Ratanatharathorn V, Nash RA, Przepiorka D (1998). Phase III study comparing methotrexate and tacrolimus (prograf, FK506) with methotrexate and cyclosporine for graft-versus-host disease prophylaxis after HLA-identical sibling bone marrow transplantation. Blood.

[9] Busca A, Saroglia EM, Lanino E (2000). Mycophenolate mofetil (MMF) as therapy for refractory chronic GVHD (cGVHD) in children receiving bone marrow transplantation. Bone Marrow Transplant.

[10] Stewart BL, Storer B, Storek J (2004). Duration of immunosuppressive treatment for chronic graft-versus-host disease. Blood.

